# Dual-mode temperature monitoring using high-performance flexible thermocouple sensors based on PEDOT:PSS/CNTs and MXene/Bi_2_Se_3_

**DOI:** 10.1038/s41378-025-00867-w

**Published:** 2025-02-25

**Authors:** Baichuan Sun, Gaobin Xu, Zhaohui Yang, Cunhe Guan, Xu Ji, Shirong Chen, Xing Chen, Yuanming Ma, Jianguo Feng

**Affiliations:** Micro Electromechanical System Research Center of Engineering and Technology of Anhui Province, School of Microelectronics, Hefei University of Technology, Hefei, Anhui 230009 People’s Republic of China

**Keywords:** Electrical and electronic engineering, Sensors

## Abstract

Due to the limited thermoelectric (TE) performance of polymer materials and the inherent rigidity of inorganic materials, developing low-cost, highly flexible, and high-performance materials for flexible thermocouple sensors (FTCSs) remains challenging. Additionally, dual-mode (contact/non-contact) temperature monitoring in FTCSs is underexplored. This study addresses these issues by using p-type (PEDOT:PSS/CNTs, 2:1) and n-type (MXene/Bi_2_Se_3_, 2:1) TE materials applied via screen printing and compression onto a PPSN substrate (paper/PDMS/Si_3_N₄). The resulting FTCSs exhibit excellent TE properties: electrical conductivities of 61,197.88 S/m (n-type) and 55,697.77 S/m (p-type), Seebeck coefficients of 39.88 μV/K and -29.45 μV/K, and power factors (PFs) of 97.66 μW/mK² and 55.64 μW/mK², respectively. In contact mode, the sensor shows high-temperature sensitivity (*S*_*T*_ = 379.5 μV/°C), a broad detection range (20-200 °C), high resolution (~0.3 °C), and fast response (~12.6 ms). In non-contact mode, it maintains good sensitivity (*S*_*Tmax*_ = 52.67 μV/°C), a broad detection range, high resolution (~0.8 °C), and even faster response (~9.8 ms). The sensor also demonstrates strong mechanical durability, maintaining stable performance after 1000 bending cycles. When applied to dual-mode temperature monitoring in wearable devices and lithium batteries, the FTCS shows high accuracy and reliability compared to commercial K-type thermocouples, indicating significant potential for advanced medical monitoring systems and smart home technologies.

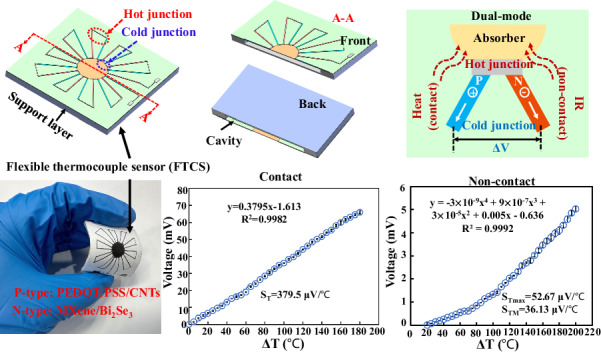

## Introduction

Temperature monitoring is crucial in various fields such as industry, medicine, and environmental monitoring, ensuring the normal operation of equipment, safety, and accurate medical diagnostics^1–3^. Thermocouple sensors, as passive devices used for measuring temperature, are widely used due to their high sensitivity and stability. However, traditional bulk thermocouples, with their rigid structures, face limitations in flexible and embedded applications, reducing measurement accuracy and potentially causing sensor damage^4,5^. In contrast, flexible thermocouple sensors (FTCSs) offer good lightweight, fast response speed, and better adaptability to complex shapes and dynamic environments, enabling real-time and precise temperature measurements^6–10^

Thermoelectric (TE) materials are crucial components of FTCSs due to their ability to convert heat directly into electricity^11^. The efficiency of this heat-to-electricity conversion is determined by the figure of merit *ZT*, given by ${ZT}=S{{\rm{\sigma }}}^{2}T/{\rm{\kappa }}$, where *S* is the Seebeck coefficient, *σ* is the electrical conductivity, *T* is the absolute temperature, and κ is the thermal conductivity^12^. The *Sσ*^2^ is known as the power factor (PF), which directly impacts the performance of TE materials^13^. While traditional inorganic metal materials, such as Bismuth Telluride (Bi_2_Te_3_)^14^, Lead Telluride (PbTe)^15^, and Bismuth Selenide (Bi_2_Se_3_)^16^ offer high TE efficiency, their application is often limited by their high cost, intrinsic rigidity, and environmental concerns. In addition to inorganic materials, TE polymers (e.g., polyaniline (PANI)^17^ and poly(3,4-ethylenedioxythiophene): polystyrenesulfonate (PEDOT:PSS)^18,19^) and inorganic nanomaterials (e.g., carbon nanotubes (CNTs)^20^ and MXene^21^) are promising due to their low cost, flexibility, and reduced toxicity. Polymers have relatively higher Seebeck coefficients but lower electrical conductivity, while nanomaterials show the opposite. Despite these advantages, both generally show TE performance that is an order of magnitude lower than traditional inorganic materials^22^.

A promising strategy to enhance TE performance involves the development of advanced composites by integrating various types of TE materials. A filler with a high Seebeck coefficient for n-type/p-type materials is added to the corresponding n-type/p-type materials. For example, Kim et al.^23^ significantly enhanced the Seebeck coefficient of PEDOT:PSS by incorporating p-type SnSe nanosheets (NSs), increasing it from 23 μV/K to approximately 200 μV/K, and achieved a *ZT*, of 0.32 with 20 wt% SnSe NSs. Cho et al.^24^ combined p-type CNTs with PEDOT:PSS and fabricated a TE generator using a wet-spinning process, resulting in a high PF of 83.2 ± 6.4 μW/mK². Chen et al.^25^ incorporated SnS nanobelts as the inorganic component in PEDOT:PSS, creating a novel TE material with a high PF of 27.8 μW/mK² at room temperature. Nevertheless, while research predominantly targets p-type polymers, there is less focus on n-type materials^26,27^. The performance of FTCSs primarily depends on the difference in PF between the n-type and p-type electrodes; the greater the difference, the better the performance. Therefore, finding suitable n-type and p-type materials and investigating their properties is crucial. Furthermore, current methods for improving n-type materials are both complex and costly. For instance, techniques such as using the solvothermal-assisted vacuum filtration method to create free-standing TE films from CNTs and Bi_2_Se_3_^27^, or combining the sol-gel method, magnesiothermic reduction, and liquid-solid phase reaction to synthesize n-type TE nanofibers from magnesium silicide (Mg_2_Si) and CNTs^28^, are employed. However, these methods often produce materials that are difficult to deposit or print on flexible substrates, and they also have limitations in terms of their application range and utility. Therefore, to simplify fabrication, reduce costs, and enhance the accuracy and sensitivity of FTCSs, it is crucial to develop high-performance TE materials compatible with common fabrication processes.

In this study, we developed a novel FTCS using a combination of p-type TE materials (PEDOT:PSS/CNTs) and n-type TE materials (MXene/Bi_2_Se_3_ NSs), fabricated through screen printing and compression techniques on a PPSN (paper/PDMS/Si_3_N₄ nanoparticles) substrate. The synthesized n-type and p-type TE materials demonstrate remarkable electrical conductivity, high Seebeck coefficients, and superior PFs. Notably, their liquid-state formulation enables their deposition through scalable techniques such as screen printing. This characteristic not only facilitates uniform material application but also underscores their suitability for the efficient fabrication of FTCS. The PPSN substrate offers excellent deformation resistance and high-temperature durability^29^. The structure of the FTCS is inspired by silicon-based IR thermoelectric detectors^30^, aiming to achieve the functionality of rigid thermocouple devices on flexible substrates. Moreover, similar designs have been utilized in flexible thermoelectric generators^31^ and sensors^32^. This novel FTCS design not only reduces costs and simplifies the fabrication process but also achieves outstanding thermoelectric performance for contact-mode temperature measurements, including high-temperature sensitivity (*S*_*T*_ = 379.5 μV/°C), a broad detection range (20-200 °C), a high detection resolution (~0.3 °C), and a fast response time (~12.6 ms). Additionally, the sensor utilizes CNTs (with good thermal conductivity, superior mechanical flexibility, and stable chemical performance) as an absorber, enabling both contact and infrared (IR) non-contact temperature monitoring. In non-contact detection, it achieves an even faster response time (~9.8 ms), high resolution (~0.8 °C), and relatively high sensitivity (*S*_*Tmax*_ = 52.67 μV/°C). Notably, there has been limited research on dual-mode (contact/non-contact) temperature detection in the field of flexible electronics, with most studies focusing on single-mode detection, primarily contact-based methods. Additionally, flexible non-contact detection has primarily been explored in the field of photoelectric detection^33,34^. The dual-mode FTCS developed in this study not only preserves the high precision and stability of contact temperature measurements but also offers enhanced application flexibility and faster response times with non-contact measurements based on Seebeck effect, paving the way for advancements in smart wearable devices and advanced medical monitoring.

## Materials and methods

### Materials and instruments

Whatman No.1 filter paper was purchased from Whatman Company, UK. PDMS (Sylgard 184) was purchased from Dow Corning, USA. Carbon nanotubes (CNTs), MXene, PEDOT:PSS, and Si_3_N₄ nanoparticles were acquired from Beike 2D Materials Co., Ltd. Bi_2_Se_3_ NSs was purchased from Zhongnuo New Materials, Beijing, China. Silane coupling agent (SCA) and acetic acid were obtained from Dongguan Shanyi Plastics Co., Ltd. The screen-printing machine was sourced from Hunan Deyun Printing Equipment Co., Ltd. Magnetic/electric stirrers, ultrasonic disruptor, constant temperature heating table, and vacuum drying oven were purchased from Lichen Technology Co., Ltd. CCD and microscope were obtained from Beijing Lepu Technology Co., Ltd. The thermostatic bath (HMDC2006) was acquired from Jiangsu Hengmin Instrument Manufacturing Co., Ltd. Lithium batteries (LiBs) and battery capacity testers were purchased from Anhui Sanuo Electronic Technology Co., Ltd. The oscilloscope (SDS1202X-C), semiconductor analyzer (Keithley 2450), DC power supply (GPD-4303S), and digital multimeter (GDM-8261A) were purchased from Future Electronic Technology Co., Ltd. The amplifier was acquired from Kangwei Technology Co., Ltd., the optical chopper (OE3001) from Guangzhou Science Instrument & Electronics Technology Co., Ltd., and the blackbody radiation source (HFY-205b) from Shanghai Biaozhuo Scientific Instruments Co., Ltd.

### Materials and sample preparation

Before fabricating the FTCSs, several pre-treatment steps are required. PDMS and the curing agent are mixed in a 10:1 ratio at room temperature. Si_3_N₄ nanoparticles are chemically modified with a silane coupling agent (SCA) to form chemical bonds with PDMS, ensuring a strong connection between the PPSN substrate materials^29^. Filter paper is dried in a vacuum oven to ensure proper adhesion between PDMS and paper, and to prevent moisture from impacting substrate performance. Bi_2_Se_3_ NSs is pre-screened to maintain a relatively uniform particle size (45 μm). Due to the high viscosity of PDMS, PDMS and Si_3_N₄ are mixed using electric stirring, while the p-type electrode materials (PEDOT:PSS and CNT solution) and the n-type electrode materials (MXene and Bi_2_Se_3_ NSs) are mixed using magnetic stirring to ensure a homogeneous blend suitable for use, as shown in Fig. S1 (Supplementary information).

### The fabrication of FTCS

**Fig. 1:**
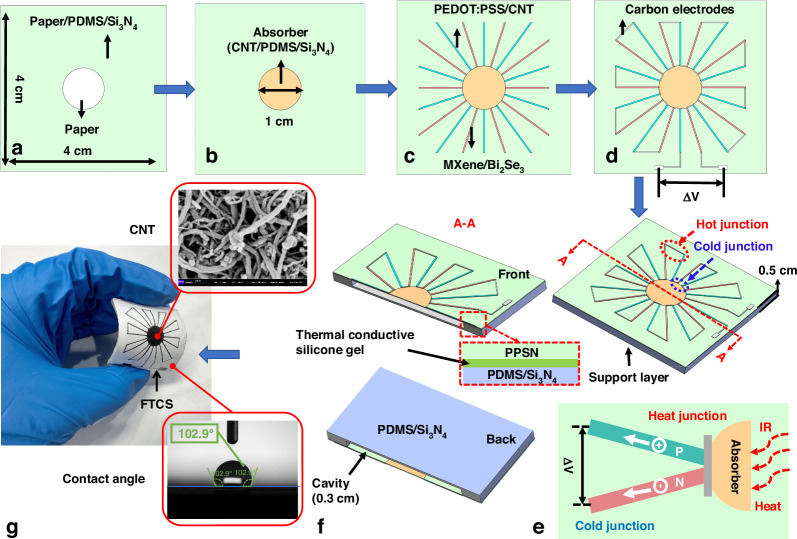
Schematic and actual images of FTCS fabrication. **a** First printing of PDMS/Si_3_N₄; **b** Printing of the absorber and second printing of PDMS/Si_3_N₄; **c** Printing of n-type/p-type electrodes; **d** Printing of carbon electrodes and third printing of PDMS/Si_3_N₄; **e** 3D structure schematic of FTCS and working principle schematic; **f** Schematic of the FTCS cross-section; **g** Actual image of FTCS

The FTCS comprises four main components: the flexible substrate, absorber, electrodes, and supporting layer, as illustrated in Fig. 1. The thermocouples are arranged in a linear structure with square spacing around the circular absorber. The fabrication primarily involves screen printing and compression.

The substrate preparation begins with designing the pattern using AutoCAD software and creating a corresponding screen for printing. As shown in Fig. 1a, the PDMS/Si_3_N₄ mixture is screen-printed onto a paper substrate (4 × 4 × 0.02 cm³), leaving a circular pattern for the absorber layer (*R* = 1 cm). The printed substrate is then heated at 80 °C in a vacuum-drying oven for 1 h. To ensure proper material adhesion, the printing is performed with applied pressure and repeated 10 times, followed by compression to enhance performance. The absorber is fabricated by screen printing CNTs onto the circular area, followed by drying (Fig. 1b). To prevent CNTs oxidation and maintain substrate integrity, a second layer of PDMS/Si_3_N₄ mixture is printed over the circular area, heated at 80 °C for 1 h, and compressed. Subsequently, the n-type and p-type electrodes were sequentially printed using a screen printing technique: blue lines in Fig. 1c represent the p-type PEDOT:PSS/CNTs mixture (width=3 mm), while red lines represent the n-type MXene/Bi_2_Se_3_ mixture. Carbon electrodes are used for connections, as shown in Fig. 1d. Finally, the entire device is coated with a PDMS/Si_3_N₄ mixture to cover all electrode patterns, preventing material oxidation, detachment, and contamination, thus ensuring optimal device performance.

In addition to the flexible substrate supporting the thermocouple, a second support layer with a cavity structure is used to reduce thermal flux loss and enhance the TE effect, forming a flexible 3-dimensional (3D) structure^35^. This layer, made from PDMS/Si_3_N₄, is produced using a mold casting method with a mold created through 3D printing technology, as depicted in Fig. 1e–g. To improve the integration of the supporting layer and facilitate heat dissipation, thermal conductive silicone gel is applied at the connection points (Fig. 1f). Figure 1g shows the scanning electron microscope (SEM) image of CNTs, which exhibit a tubular morphology. The entire sensor demonstrates hydrophobic properties with a contact angle of 102.9°, indicating strong hydrophobicity (contact angle > 90°).

### The principle of temperature detection

The working principle of FTCSs is based on the Seebeck effect, as shown in Fig. 1e. When the sensor absorbs the heat or IR radiation, the temperature at the hot junction of the thermocouple connected to the absorber rises, while the cold junction remains at the surrounding temperature. This temperature difference (*ΔT*) causes carriers within the conductor to move from the hot junction to the cold junction under the gradient, accumulating and forming a potential difference (*ΔV*)^34^. The *ΔV* can be expressed as follows:

1 $$\varDelta V=N{\alpha }_{{ab}}\varDelta T$$

where *N* is the number of thermocouples, and *α*_*ab*_ is the Seebeck coefficient difference between the two thermocouple materials. In contact temperature measurements, the absorber directly absorbs heat without energy conversion, so the *ΔV* is proportional to *ΔT*. However, in IR temperature detection, the *ΔT* is still generated by the hot and cold ends, but its expression is determined by the IR radiation intensity^30,34^, as described by the following equations:

2 $$\varDelta T=\frac{{\rm{\eta }}{P}_{0}}{{G}_{th}}$$

3 $${P}_{0}={\varPhi }_{0}{A}_{d}=\frac{\sigma {C}_{r}{\varepsilon }_{1}\left({T}_{1}^{4}-{T}_{0}^{4}\right){A}_{s}{A}_{d}}{\pi {d}_{0}^{2}}$$

where P_0_ is the IR radiation power, η is the absorptivity of the absorber, *G*_*th*_ is the total thermal conductance of the sensor, *Φ*_0_ is the IR radiation power density, *A*_*d*_ is the absorption area of the detector, *σ* is the Stefan-Boltzmann constant, *C*_*r*_ is the root-mean-square conversion factor, *ε*_1_ and *A*_*s*_ is the emissivity and the area of the radiation source, respectively, *d*_0_ is the distance between the surface of the radiation source and the detector, *T*_1_ is the temperature of the blackbody radiation source, and *T*_0_ is the ambient temperature. Thus, it can be seen that in non-contact IR temperature detection, the relationship between the *ΔV* and temperature follows a fourth-degree polynomial.

## Results and discussion

### Simulation analysis

**Fig. 2:**
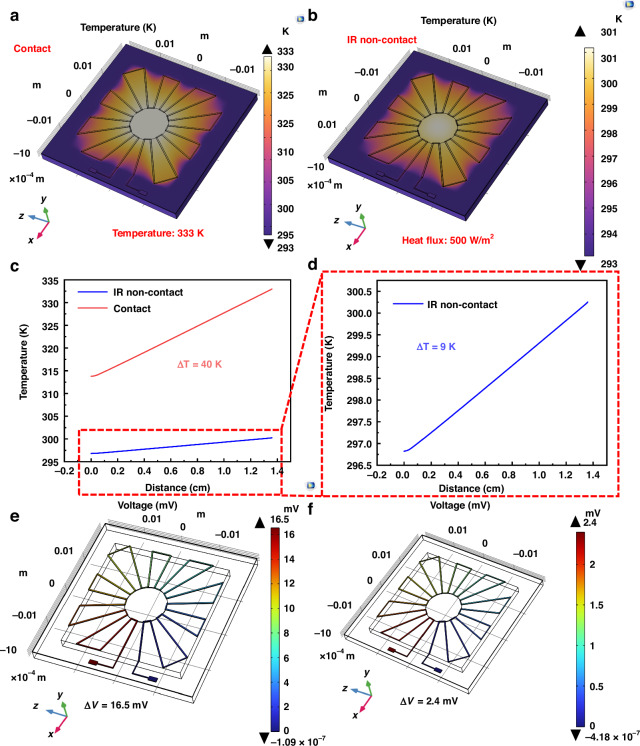
Simulation analysis of contact and IR non-contact temperature detection. **a** Temperature distribution inside the FTCS under contact mode; **b** Temperature distribution inside the FTCS under IR non-contact mode; **c**, **d** Comparison of the *ΔT* between the hot and cold ends in both modes; **e** Potential distribution inside the FTCS under contact mode; **f** Potential distribution inside the FTCS under IR non-contact mode

Prior to the experimental analysis, a TE simulation of the FTCS was conducted using COMSOL Multiphysics (Burlington, MA, USA), with the model created in SolidWorks (Waltham, MA, USA). As shown in Fig. 2, TE simulations were conducted for both contact and IR non-contact temperature detection modes. The boundary conditions of the FTCS were set based on actual testing and conditions. The temperature was set to 333.15 K (60 °C), the sensor terminal to 293.15 K (20 °C), and the heat flux of radiation source to 500 W/m². Additionally, the FTCS terminus was grounded, and the entire sensor was in a static TE state.

As illustrated in Fig. 2a, b, a clear *ΔT* exists between the hot junction and the cold junction, where bright yellow represents higher temperature, and purple represents lower temperature. This indicates that the main heat conduction occurs within the FTCS and transfers to the bottom of the supporting layer. Figure 2c, d depict a *ΔT* of 40 K in contact detection (in an ideal simulation) and 9 K in non-contact detection, with the latter difference arising from the conversion of radiative energy into heat energy, as explained by Eq. (2). According to the Seeback effect, the *ΔT* causes a *ΔV* within the sensor. Figure 2e, f show the potential distribution in both scenarios, with a *ΔV* of 16.5 mV in contact detection and 2.4 mV in non-contact detection. This not only confirms the presence of the TE effect but also highlights the well-designed rationality of the FTCS for dual-mode temperature detection.

### Characterization and performance analysis of TE materials

#### Characterization analysis of TE materials

**Fig. 3:**
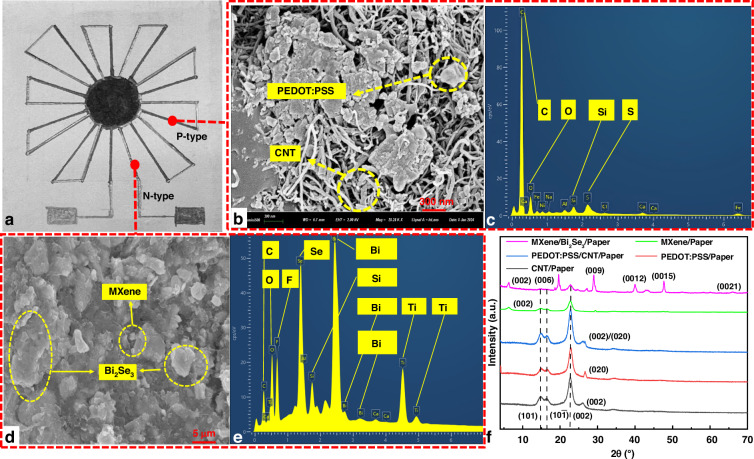
Characterization analysis of n/p-type electrode materials in FTCS. **a** Front view of FTCS; **b** SEM image of p-type TE materials; **c** EDS analysis of p-type electrodes; **d** SEM image of n-type TE materials; **e** EDS analysis of n-type electrodes; **f** XRD comparison of different materials

To more intuitively display the composition and interrelationship of n-type/p-type TE electrode materials, SEM, energy dispersive spectroscopy (EDS), and X-ray diffraction (XRD) were used for characterization analysis. Figure 3a shows the front view of the FTCS, which features 10 pairs of n-type and p-type electrodes. The p-type electrode consists of PEDOT:PSS and CNTs, with its microstructure illustrated in Fig. 3b. PEDOT:PSS is represented by the block-like structure, while CNTs is depicted as a linear tubular structure. These two materials are randomly interwoven. Figure 3c shows the elemental composition, where C and O elements are predominant due to the presence of PEDOT:PSS, CNTs, and the substrate. S, a key element of PEDOT:PSS, confirms its presence. The presence of Si is attributed to the PDMS in the substrate. The n-type electrode consists of MXene and Bi_2_Se_3_ nanosheets, both appearing as sheet-like structures under the microscope, as shown in Fig. 3d. Bi_2_Se_3_ exhibits a larger sheet structure compared to MXene. The MXene used in this experiment is Ti_3_C_2_T_x_, composed of Ti, C, T_x_ (F and O atoms), and H. These components are identifiable in the elemental distribution shown in Fig. 3e. Bi_2_Se_3_ mainly consists of Bi and Se, which are clearly visible in the elemental distribution.

Additionally, to more clearly compare the composition and interrelationships of the materials, we compared the crystalline structures of the mixed and individual materials on the substrate, as shown in Fig. 3f. Notably, due to the presence of cellulose nanofibers in the substrate, diffraction peaks corresponding to the (101), (10$\bar{1}$), and (002) crystal planes appear at approximately 2θ = 14.9°, 16.5°, and 22.8°, respectively^29^. For the p-type TE material, a diffraction peak corresponding to the (002) crystal plane, indicating the reflection peak of C, can be observed at approximately 26.2°, confirming the presence of CNTs^36^. In the XRD pattern of PEDOT:PSS, a diffraction peak corresponding to the (020) crystal plane, indicating the π–π stacking of the PEDOT thiophene ring, can be observed at approximately 25.8°^37^. Therefore, in the mixture of the two materials, these crystal planes are observed, confirming the presence of each component and demonstrating successful mixing. For the n-type TE materials, the MXene exhibits a diffraction peak corresponding to the (002) crystal plane at approximately 6.9°, characteristic of MXene, reflecting its interlayer structure^38^. Moreover, this peak is also observed in the MXene/Bi_2_Se_3_ mixture in Fig. 3f. However, with the addition of Bi_2_Se_3_, the reflection peak shifts to 4.6°, indicating that the Bi_2_Se_3_ NSs increased the interlayer spacing in MXene. This observation aligns with previous reports^29^^,39^^,40^. Furthermore, in the XRD pattern of the mixture, diffraction peaks corresponding to the (006), (009), (0012), (0015), and (0021) crystal planes of Bi_2_Se_3_ are observed at approximately 2θ = 18.5°, 28.1°, 37.2°, 47.8°, and 70.6°, respectively, confirming the successful synthesis of Bi_2_Se_3_ NSs ^41^.

#### TE performance analysis of materials

**Fig. 4:**
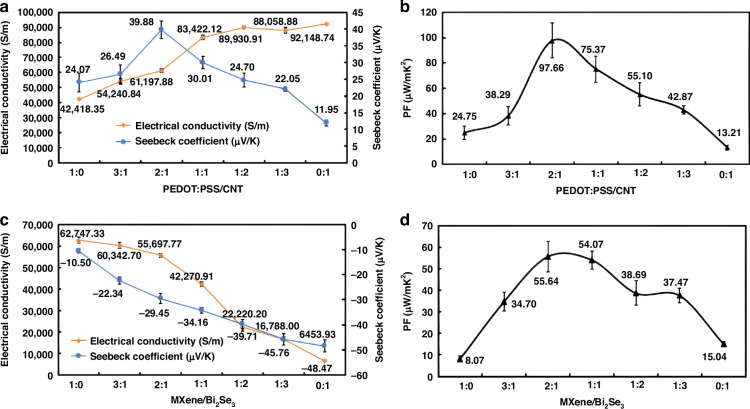
Performance Analysis of Thermoelectric Materials. **a** Comparison of electrical conductivity and Seebeck coefficient for different ratios of PEDOT:PSS/CNTs; **b** Comparison of PF for different ratios of PEDOT:PSS/CNTs; **c** Comparison of electrical conductivity and Seebeck coefficient for different ratios of MXene/Bi_2_Se_3_; **d** Comparison of PF for different ratios of MXene/Bi_2_Se_3_. (*n* = 5, Mean ± SD)

To compare the overall performance of the composite materials, this study examined the conductivity, Seebeck coefficient, and PF at different mixing ratios. The goal was to identify the optimal mixing ratio for TE materials to enhance their performance. The performance of the TE materials, as shown in Fig. 4, was methodically observed at room temperature. Figure 4a shows that the electrical conductivity of PEDOT:PSS on the PPSN substrate is approximately 42,418.35 S/m. (Note that in Fig. 4 and all subsequent figures, the error bars represent the standard error, calculated from the mean and standard deviation (SD) of each experiment. Each experimental set in this study was repeated five times (*n* = 5), and all data are presented as Mean ± SD.) As the CNTs content increases, the electrical conductivity of the composite material gradually enhances, showing an upward trend. When only CNTs is present, the conductivity reaches a maximum of around 92,148.74 S/m, demonstrating that CNTs has superior conductivity compared to PEDOT:PSS. The superior conductivity of CNTs is primarily due to their ability to conduct electricity through conduction band electrons. Additionally, their fibrous structure effectively links the conductive pathways within PEDOT:PSS, further enhancing the overall conductivity of the mixture. In contrast, the Seebeck coefficient of PEDOT:PSS is 24.07 μV/K. As the CNTs content increases, the Seebeck coefficient of the composite initially rises and then decreases, reaching a peak value of 39.88 μV/K at a 2:1 ratio of PEDOT:PSS to CNTs, with the corresponding electrical conductivity reaching 61,197.88 S/m. However, when only CNTs is present, the Seebeck coefficient drops to 11.95 μV/K. This trend occurs because the Seebeck coefficient of PEDOT:PSS is higher than that of CNTs. When the CNTs content is low, PEDOT:PSS dominates in the mixture, and the structure of CNTs can optimize charge transfer between polymers, increasing the contribution of high-energy carriers. Furthermore, strong interfacial interactions, such as π-π conjugation and van der Waals forces, exist between the polymer chains of PEDOT:PSS and CNTs, which further enhance carrier transport within the composite films^42,43^. This leads to an improved Seebeck coefficient. However, as the CNTs content increases, CNTs becomes dominant in the mixture, resulting in a decrease in the Seebeck coefficient. Therefore, as shown in Fig. Fig. 4b, the optimal TE performance is achieved when the PEDOT:PSS to CNTs ratio is 2:1, yielding a PF of 97.66 μW/mK². Furthermore, the thermal conductivity (κ) of the composite material was measured to be approximately 1150 W/m·K at 25 °C. Based on this measurement, the corresponding *ZT* value was calculated to be 2.53 × 10⁻⁵.

For the n-type TE materials, as shown in Fig. 4c, MXene exhibits an electrical conductivity of 64,301.33 S/m and a Seebeck coefficient of -10.5 μV/K. The negative Seebeck coefficient indicates that electrons are the primary contributors to charge transport^44^. As the content of Bi_2_Se_3_ NSs increases, the electrical conductivity decreases, while the Seebeck coefficient increases. When only Bi_2_Se_3_ is present, the conductivity drops to 6,453.93 S/m, but the Seebeck coefficient rises to -48.47 μV/K. Several factors contribute to this phenomenon: First, the two-dimensional layered structure of MXene exhibits excellent metallic conductivity, while Bi_2_Se_3_ has moderate conductivity. As the Bi_2_Se_3_ content increases, the carrier concentration and mobility decrease, leading to a reduction in conductivity^27^. Second, the interlayer gaps in Bi_2_Se_3_ NSs significantly reduce their electrical conductivity compared to Bi_2_Se_3_ films. When incorporated between MXene layers, these gaps widen the interlayer spacing of MXene, as shown by previous XRD analysis of the composite, further reducing conductivity. Additionally, due to high electron of MXene mobility and carrier density, its Seebeck coefficient is relatively low. However, since Bi_2_Se_3_ has a higher Seebeck coefficient, blending it with MXene improves the overall Seebeck coefficient of the material. Similarly, calculations show that the optimal PF is achieved at a 2:1 ratio of MXene to Bi_2_Se_3_, resulting in a maximum PF of 55.64 μW/mK² (Fig. 4d), with a corresponding electrical conductivity of 55,697.77 S/m and a Seebeck coefficient of -29.45 μV/K. Similarly, the thermal conductivity (*κ*) of the n-type TE material at 25 °C was measured to be 28 W/m·K. Based on this measurement, the calculated *ZT* value for this material is 5.93 × 10⁻⁴.

To clearly demonstrate the characteristics of the n-type and p-type TE materials obtained in this study at room temperature, we compared them with previously reported TE composites, as shown in Tables 1 and 2. The tables reveal that the performance of the p-type PEDOT:PSS/CNTs mixture and the n-type MXene/Bi_2_Se_3_ mixture is comparable to, or even superior to, most other reported composites. Moreover, these materials are characterized by simple processing and strong practicality, which clearly demonstrates the superiority of the n-type and p-type materials proposed in this study. In summary, the synthesized n-type and p-type TE materials demonstrate excellent electrical conductivity, high Seebeck coefficients, and favorable PFs. Their high compatibility with flexible substrates further ensures that they meet the fabrication and performance requirements for FTCS.

**Table 1:** Comparison of p-Type TE materials performance at room temperature

Materials	Method	*σ*(S/m)	*S*(μV/K)	PF(μW/mK²)	Ref.
PEDOT:PSS/SnSe nanosheets (20 WT%)	Hydrothermal lithium-intercalation/exfoliation	~32,000	~110	~387	^23^
PEDOT:PSS/CNT	wet-spinning	97,900 ± 23,200	29.3 ± 2.1	83.2 ± 6.4	^24^
PEDOT:PSS/SnS nanobelt	hydrothermal/ ultrasonication mixing	697.5 ± 4.6	19.1 ± 0.4	27.8 ± 0.5	^25^
PEDOT:PSS/CNT/PCL	filtration/hot pressing	~1581	~35	~1.9	^43^
PEDOT:PSS/rGO/CNTs	thermal reduction/comounding	~178811	~15.87	~45.03	^48^
PEDOT:PSS/CNT/graphene	in situ polymerization	~293.6	~18.9	~0.105	^49^
PEDOT:PSS/CNT	Magnetic/ultrasonic mixing	~61197.88	~39.88	~97.66	this work

**Table 2:** Comparison of n-Type TE materials performance at room temperature

Materials	Method	*σ*(S/m)	*S*(μV/K)	PF(μW/mK²)	Ref.
Bi_2_Se_3_/SWCNTs	solvothermal	~29270	~−42.4	~52.7	^27^
Mg_2_Si/CNTs	sol-gel method/ magnesiothermic reduction/liquid-solid phase reaction	~0.98	~−30	~8.94 × 10^−3^	^28^
Bi_2_Se_3_/PVDF	solvothermal	~5100	~−80	~32.6	^50^
Cu_0.1_Bi_2_Se_3_/PVDF	solvothermal	~14600	~−84	~103.2	^51^
Bi_2_Se_3_/MWCNTs	chemical deposition	~530	~−85	~0.4	^52^
Bi_2_Te_3_/SWCNTs	solvothermal	~24460	~−39.1	~32.3	^53^
Sb_2_Te_3_/SWCNTs	solvothermal	~40000	~−40	~58.9	^54^
Bi_2_Se_3_/MXene	Magnetic/ultrasonic mixing	~55697.77	~−29.45	~55.64	this work

### Performance analysis of FTCS in the contact mode temperature monitoring

**Fig. 5:**
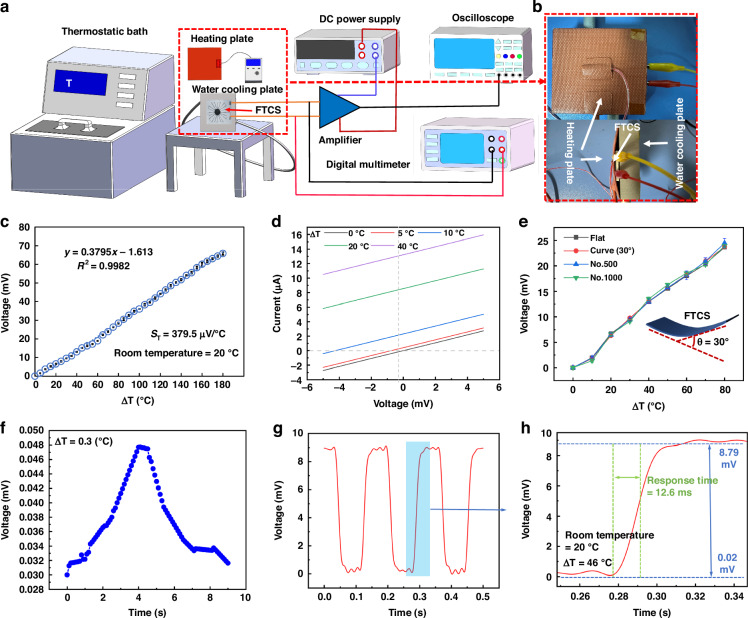
Temperature sensitivity testing of FTCS in contact mode. **a**, **b** Schematic and real images of the experimental platform; **c** Voltage vs. temperature difference curve of the FTCS across a temperature range of 20–200 °C; **d** Linear relationship of I-V curves at different temperatures; **e** Voltage vs. temperature difference curve of the sensor under different conditions; **f** Output voltage of sensor at *ΔT* = 0.3 °C; **g**, **h** Response time of FTCS at *ΔT* = 46 °C. (n = 5, Mean ± SD)

To thoroughly assess the temperature sensing performance of the FTCS in contact mode and to showcase the practical application of TE materials, this study evaluates the FTCS across a temperature range of 20 to 200 °C (in 5 °C increments) under varying conditions, including flat and curved surfaces, as well as repeated bending deformation. The experimental setup is shown in Fig. 5a, b. The setup includes a thermostatic bath, water cooling plate, heating plate, amplifier, digital multimeter, semiconductor analyzer, DC power supply, and oscilloscope. To maintain a constant test temperature, the experimental setup was maintained at a constant temperature of 20 °C. A water circulation cooling system was used, with the back of the FTCS attached to a water-cooling plate to keep the cold end temperature stable (20 °C). The schematic in Fig. S2 (Supplementary information) shows that the presence of a cavity in the substrate (which reduces heat conduction) directs the cooling cycle to the cold end region, ensuring the temperature at the cold end remains constant. A heating plate was then employed as a stable heat source to test the performance of sensor, as shown in Fig. 5b. As seen in the curve in Fig. 5c, the voltage of the FTCS increases with temperature rising, showing a linear relationship with an R² value of 0.9982. This indicates that in contact mode, the sensor has stable temperature sensing performance and can accurately detect a wide range of temperatures, maintaining good temperature monitoring even at high temperatures of 200 °C. The temperature sensitivity of the FTCS is defined as ${S}_{T}=\varDelta V/\varDelta T$. From Fig. 5c, it can be seen that the sensitivity of this sensor reaches 379.5 μV/°C at *ΔT* = 180 °C, demonstrating excellent temperature sensitivity, which is superior to most reported FTCS performances, as shown in Table 3. This further demonstrates the practicality and effectiveness of the aforementioned hybrid materials. Additionally, to validate the effectiveness of the structure in this study, the performance of the single-node FTCS and the array structure was compared in the 20-100 °C range, as shown in Fig. S2a–c (Supplementary information). Both configurations demonstrated a strong linear correlation between voltage and temperature, with R² values of 0.9931 for the single-node and 0.9929 for the array. The results indicate that the array significantly enhanced measurement stability by summing the electromotive force across multiple nodes, resulting in higher sensitivity compared to the single-node FTCS, which had a sensitivity of only around 33.9 μV/°C. Notably, performance tests were also conducted on single-node FTCSs made from PEDOT:PSS with MXene and CNT with MXene. However, within this temperature range, it was difficult to detect signals, or they exceeded the detection range of the instruments used in this study. This is attributed to the low Seebeck coefficient of MXene and CNT, further highlighting the superiority of the materials used in this research. Fig. S4a, b) (Supplementary information) illustrate the principle of increasing the output electromotive force caused by the array, where the single-node thermocouple generates a voltage (U) between the hot and cold junctions due to the *ΔT*, and the array, consisting of N pairs of thermocouples connected in series, produces a voltage that is N times higher than a single pair under ideal conditions^45^. Notably, the increased sensitivity is not solely due to higher voltage but is also closely related to structural design optimization and improved signal processing methods^30,46^, highlighting the clear advantages of using an array configuration for temperature detection. Moreover, the I-V characteristic curve in Fig. 5d shows a linear relationship between voltage and current, indicating that the sensor has perfect ohmic contact properties. Moreover, as the *ΔT* (5 °C, 10 °C, 20 °C, 40 °C) varies, the I-V curves of the FTCS remain relatively parallel, suggesting that temperature has little effect on the resistance of the entire device.

**Table 3:** Comparison of sensitivity and detection ranges for different FTCS

Materials	S_T_ (μV/°C)	Range (°C)	Response time (ms)	Ref.
Graphene-FeCl_3_/Ag	~281.46	20–60	~780	^32^
Bi_0.5_Sb_1.5_Te/ Bi_2_Te_2.7_Se_0.3_	~319	20–100	—	^55^
Platinum/In_2_O_3_	~76.5	20–100	—	^7^
Alumel (Ni/Al/Si/Mn) and chromel (Ni/Cr)	~41.2	10–110	—	^47^
Graphene oxide (GO) liquid crystals (LCs)	~12.5	~20–70	240	^56^
In_2_O_3_/ITO	~226.7	~−196–1200	4–5	^57^
MWCNTs-PEDOT: PSS/Cu	~220	0–100	57.6	^58^
PEDOT:PSS-CNT/MXene-Bi_2_Se_3_	~376.1	20–220	12.6	this work

Additionally, to illustrate that the FTCS can be applied beyond flat surfaces, temperature testing was performed with a bending deformation angle (θ) of 30°. As shown in Fig. 5e, despite the deformation, the voltage versus temperature difference curve remains consistent with that of the flat structure. This demonstrates the suitability of sensor for curved surfaces and its resistance to bending deformation, a property attributed to the deformation-resistant characteristics of the PPSN substrate^29^. To further evaluate the durability of sensor, bending deformation tests were performed 500 and 1000 times. Figure 5e demonstrates that even after repeated bending, the performance remains stable, confirming its excellent mechanical durability. Additionally, as shown in Fig. 5f, the FTCS can detect temperature differences as small as *ΔT* = 0.3 °C, which is crucial for monitoring slight temperature variations, indicating the high-temperature resolution of sensor. This also proves that the sensor is capable of detecting subtle temperature changes, providing a reliable solution for precise temperature monitoring in daily applications. The response time is another key performance metric. For thermocouples, it is defined as the time required to reach 63.2% of a sudden temperature change^30^. We tested the response time of sensor under a temperature shock, and as shown in Fig. 5g, h, with a *ΔT* of 46 °C, the measured response time is as low as 12.6 ms, showcasing the rapid response capability of sensor. This performance compares favorably with other thermocouples reported in the literature, as shown in Table 3.

### Performance analysis of FTCS in the IR non-contact mode temperature monitoring

**Fig. 6:**
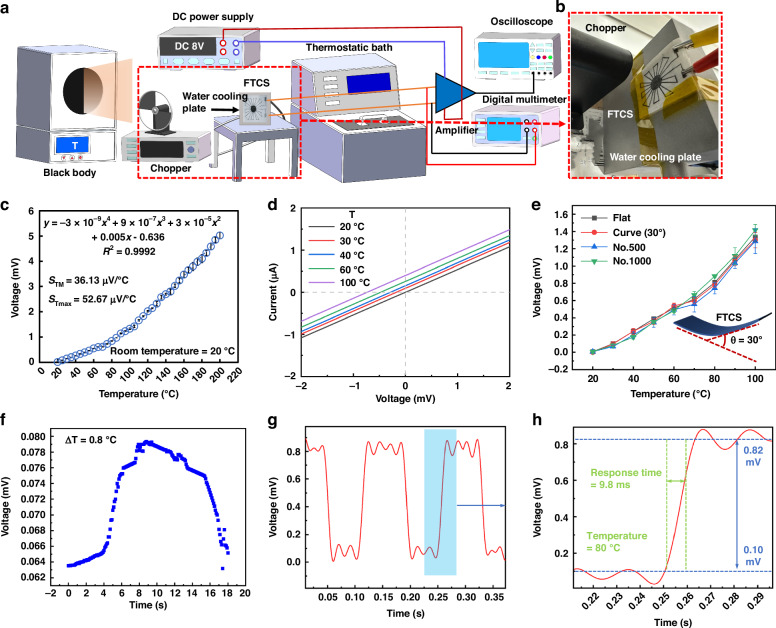
Temperature sensitivity testing of FTCS in IR non-contact mode. **a**, **b** Schematic and real images of the experimental platform; **c** Voltage vs. temperature difference curve of the FTCS across a temperature range of 20–200 °C; **d** Linear relationship of the I-V curves of sensor at different temperatures; **e** Voltage vs. temperature difference curve of the sensor under different conditions; **f** Output voltage of sensor at *ΔT* = 0.8 °C; **g**, **h** Response time of FTCS from room temperature to 80 °C. (*n* = 5, Mean ± SD)

Besides its excellent temperature detection in contact mode, the FTCS also exhibits impressive temperature detection performance in IR non-contact mode. To assess its performance, an experimental setup was created, as depicted in Fig. 6a, b. This setup includes a blackbody radiation source (IR emitter), optical chopper, thermostatic bath, water-cooling plate, amplifier, semiconductor analyzer, DC power supply, digital multimeter, and oscilloscope, with the FTCS positioned directly in front of the blackbody radiation source. The blackbody temperature was varied from 20 °C (293.15 K) to 200 °C (473.15 K) in 5 °C increments, maintaining a constant distance of 78 mm between the FTCS and the blackbody. Similarly, to maintain a constant ambient temperature, the laboratory temperature was set to a stable 20 °C. Additionally, the back of the FTCS was attached to a water-cooled plate to ensure that the cold end temperature remained constant at 20 °C.The relationship between temperature and voltage was examined under these conditions. As shown in Fig. 6c, the voltage of sensor increased with temperature, following a quartic relationship predicted by theoretical models (Eqs. (1)-(3)). The data were fitted with a quartic polynomial model, which demonstrated an excellent fit with an R² value of 0.9992. This V-T curve confirms the positive response characteristic of sensor across the temperature range, validating its capability for non-contact temperature detection. Due to the nonlinear voltage-temperature relationship under these conditions, temperature sensitivity (*S*_*T*_) was calculated for each temperature segment, yielding a mean sensitivity (*S*_*TM*_) of 36.13 μV/°C and a maximum sensitivity (*S*_*Tmax*_) of 52.67 μV/°C. This sensitivity is lower in comparison to contact mode because thermal radiation has a lower heat transfer efficiency than direct solid-to-solid conduction. Solid conduction, with its higher heat transfer capacity, enables contact measurements to more effectively capture temperature changes, leading to greater sensitivity. Despite this, the sensor demonstrates good sensitivity and greater operational flexibility, making it ideal for high-temperature, moving objects, or hazardous environments, thus addressing a need in flexible TE IR temperature monitoring. Additionally, Fig. 6d shows that the I-V curves of sensor remain relatively parallel across various temperatures (20, 30, 40, 60, 100 °C), indicating minimal impact of temperature on the resistance of sensor in non-contact mode.

Temperature tests were also performed under bending conditions, as illustrated in Fig. 6e. The results demonstrate that the temperature sensing capability of sensor remains consistent whether flat or bent at 30°, with no significant variation in the curves. The sensor continued to exhibit good temperature sensitivity in non-contact mode even after 500 and 1000 bending cycles. However, Fig. 6e shows increased data deviation compared to contact mode, suggesting higher susceptibility to environmental factors in noncontact mode. Nevertheless, Fig. 6f indicates that the sensor can detect temperature differences as small as *ΔT* = 0.8 °C in non-contact mode, reflecting its relatively high resolution. Although this resolution is lower than that in contact mode, it is still suitable for daily temperature monitoring where high precision is not critical, especially in non-emergency situations or when monitoring large areas. Additionally, Fig. 6g, h show a rapid response time of approximately 9.8 ms when the temperature increases from room temperature to 80 °C, which is faster than in contact mode. This quicker response is due to the absence of the need for thermal equilibrium, as the temperature is directly measured through IR radiation, further confirming the effectiveness of the sensor in non-contact temperature detection.

### Physical application of FTCS

#### Physical application of FTCS in wearable devices

**Fig. 7:**
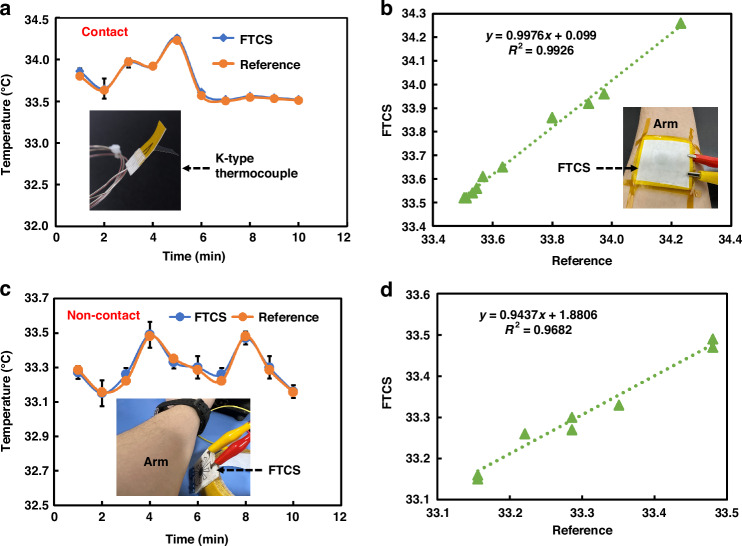
Practical application of FTCS in wearable devices. **a** Comparison of real-time temperature monitoring between FTCS and K-type thermocouple on the arm in contact mode; **b** Correlation between FTCS measurements and reference temperature in contact mode; **c** Comparison of real-time temperature monitoring between FTCS and K-type thermocouple on the arm in non-contact mode; **d** Correlation between FTCS measurements and reference temperature in non-contact mode. (*n* = 5, Mean ± SD)

Wearable devices play a crucial role in temperature monitoring, offering real-time and convenient tracking of body temperature. This capability is essential for the early detection of health anomalies and enables preventive interventions. Therefore, this study utilizes FTCS to monitor human body temperature in both contact and non-contact modes. To validate the practicality and accuracy of the FTCS developed in this study, a comparison was conducted using a standard K-type thermocouple (Ni-Cr/Ni-Si). Figure 7a shows the real-time temperature monitoring results of the FTCS and K-type thermocouple in contact mode, with both sensors attached to the arm as depicted in Fig. 7b. For comparison, the voltage from the FTCS were converted to temperature using the equation in Fig. 5c. As shown in the curve of Fig. 7a, the temperature from both the FTCS and the K-type thermocouple are consistent, maintaining approximately 33.5 °C, which aligns with the normal arm temperature of a healthy individual^29^. By comparing the reference temperature with the temperature measured by the FTCS, as illustrated in Fig. 7b, the relationship was found to be $y=0.9976x+0.099$, with an R² value of 0.9926. This high level of correlation indicates strong agreement between the two measurements, supporting the suitability of FTCS for contact-based temperature monitoring in wearable devices. This is related to the temperature resolution of 0.3 °C in contact mode, which allows for precise monitoring of small-scale temperature variations, enabling the detection of subtle abnormalities.

Additionally, Fig. 7c presents the monitoring of body temperature using FTCS in non-contact mode. The results were again compared with those from the K-type thermocouple. The trends observed in the non-contact mode were generally consistent with the reference values, although the deviations were larger compared to the contact mode. This further demonstrates that non-contact measurements are more susceptible to environmental factors. As shown in Fig. 7d, the relationship between the two measurements in non-contact mode is expressed as $y=0.9437x+1.8806$, with an R² of 0.9682. Although the R² is slightly lower than that of the contact mode, it remains above 0.95, indicating a relatively strong correlation. Notably, a slight deviation was observed between the body temperature readings from the sensor and those obtained from commercial sensors in this mode. This deviation can be attributed to the resolution of the sensor (~0.8 °C) in non-contact mode. Furthermore, the sensor, although not achieving perfect precision in non-contact body temperature monitoring, remains sufficient for general temperature monitoring applications. In particular, it can effectively detect temperature variations, such as increases or decreases, in non-emergency situations or during large-scale monitoring (e.g., fever due to illness or post-exercise). This broadens the potential applications of FTCS in this field. For instance, FTCS could be attached to different parts of the body or embedded directly into clothing to enable real-time monitoring of body temperature.

#### Physical application of FTCS in temperature monitoring of lithium battery

**Fig. 8:**
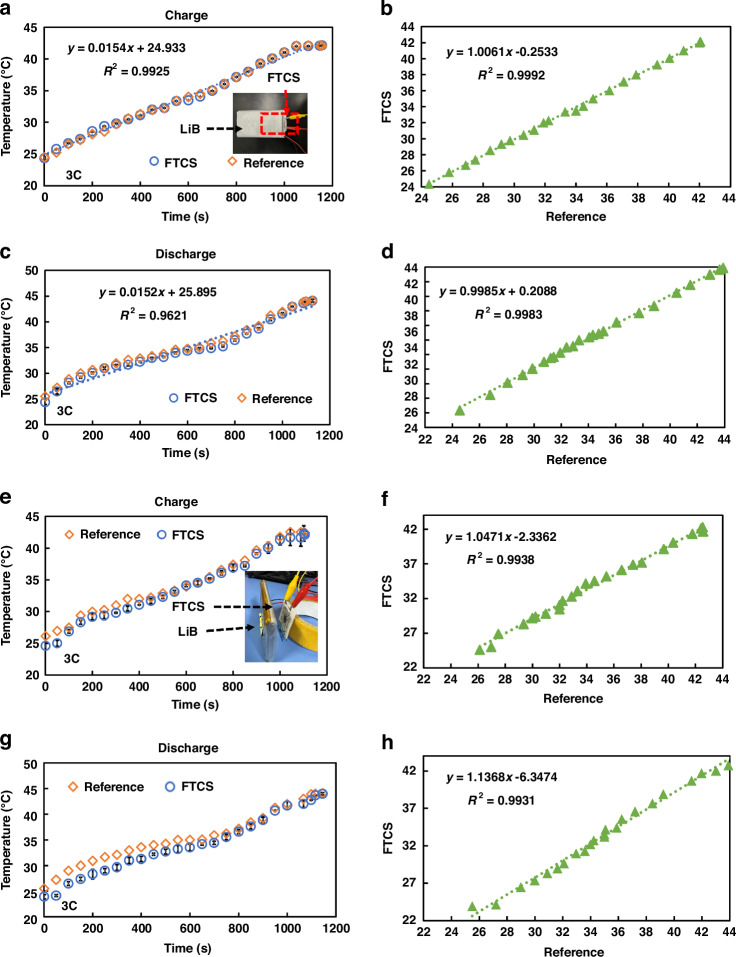
Real-time temperature monitoring of LiBs using FTCS. **a**, **b** Comparison and correlation of FTCS and K-type thermocouples in contact mode during charging; **c**, **d** Comparison and correlation in contact mode during discharging; **e**, **f** Comparison and correlation of FTCS and K-type thermocouples in non-contact mode during charging; **g**, **h** Comparison and correlation in non-contact mode during discharging. (*n* = 5, Mean ± SD)

Real-time temperature monitoring of lithium batteries (LiBs) is crucial for detecting abnormal temperature rises promptly, preventing thermal runaway, extending battery life, and ensuring safe operation of the device. Therefore, this study not only applies FTCS to wearable devices but also integrates it with LiBs for real-time temperature monitoring, as shown in Fig. 8. Similar to previous experiments, the monitoring results of the K-type thermocouple and FTCS were compared. In the contact mode, the sensors were affixed inside the LiB casing, as shown in Fig. 8a. The LiB used was a commercial type (10 Ah, 110 × 60 × 12 mm³). The battery was charged and discharged at a rate of 3 C (9 A), and temperature changes were recorded. A comparison of Fig. 8a, c) reveals that the heat generated during discharge is slightly higher than during charging. The temperature initially rises rapidly, then stabilizes, and finally increases quickly. The linear relationship between temperature and time during charging is superior to that during discharge (R² values of 0.9925 and 0.9621, respectively), consistent with previous studies^29,47^. More importantly, comparing the temperature monitoring results of FTCS and the K-type thermocouple in contact mode shows strong agreement. Correlation analyses (Fig. 8b–d) reveal that during charging and discharging, the relationships are $y=1.0061x-0.2533$ and $y=0.9985x+0.2088$, with R² values of 0.9992 and 0.9983, respectively. This indicates that the monitoring results of FTCS and the K-type thermocouple are highly consistent in this mode, demonstrating the practicality and effectiveness of FTCS for real-time temperature monitoring of LiBs.

Furthermore, the same experiments were repeated in non-contact mode, as shown in Fig. 8e–h. During charging and discharging, the temperature trends were similar to those observed in contact mode, with an initial rapid increase, followed by a stable rise, and then another rapid increase. Similarly, temperature deviations in non-contact mode were larger compared to contact mode. Nonetheless, the R² values for the temperature measurements during charging and discharging compared to the K-type thermocouple reference temperatures remained above 0.95, at 0.9938 and 0.9931, respectively. The relationships are y = 1.0471x – 2.3362 and y = 1.1368x – 6.3474. This confirms that FTCS can effectively perform non-contact temperature monitoring of LiBs while maintaining relatively high accuracy.

## Conclusion

This study developed a dual-mode FTCS based on p-type (PEDOT:PSS/CNTs) and n-type (MXene/Bi_2_Se_3_ NSs) materials, fabricated through screen printing and compression techniques. This FTCS enables both real-time contact temperature monitoring and non-contact temperature detection via the Seebeck effect. The mixed materials exhibit excellent TE properties, with electrical conductivities of 61,197.88 S/m (PEDOT:PSS/CNTs) and 55,697.77 S/m (MXene/Bi_2_Se_3_), and Seebeck coefficients of 39.88 μV/K and -29.45 μV/K, respectively. In contact mode, the sensor demonstrates excellent performance with high sensitivity (*S*_*T*_ = 379.5 μV/°C), a wide detection range (20-200 °C), high resolution (~0.3 °C), and fast response time (~12.6 ms). In non-contact mode, it maintains reasonable sensitivity (*S*_*Tmax*_ = 52.67 μV/°C), a wide detection range (20-200 °C), resolution (~0.8 °C), and a faster response time (~9.8 ms). Additionally, the sensor exhibits excellent mechanical durability, maintaining performance after 1,000 bending cycles. When applied to wearable devices and LiBs temperature monitoring, it offers high accuracy and reliability, comparable to commercial K-type thermocouples. Despite some challenges, such as sensitivity to environmental factors and lower sensitivity in noncontact mode, this dual-mode FTCS provides precise measurements and flexibility, with promising potential for applications in healthcare, industrial monitoring, and the Internet of Things.

## Supplementary information

Response time of dual-mode temperature monitoring

Supplemental Material File #1
