# Supplementary material for: Dual-mode temperature monitoring using high-performance flexible thermocouple sensors based on PEDOT:PSS/CNTs and MXene/Bi2Se3
Source: Microsyst Nanoeng. 2025 Feb 25;11:31. doi: 10.1038/s41378-025-00867-w (PMC11850902; doi:10.1038/s41378-025-00867-w)
Supplement: Supplementary file 2 — Supplemental Material File #1 [file 41378_2025_867_MOESM2_ESM.docx]

**Supplementary Information**

**Dual-Mode Temperature Monitoring Using High-Performance Flexible Thermocouple Sensors Based on PEDOT:PSS/CNTs and MXene/Bi₂Se₃**

Baichuan Sun^a^, Gaobin Xu^a^*, Zhaohui Yang ^a^,Cunhe Guan^a^, Xu Ji^a^, Shirong Chen^a^, Xing Chen^a^, Yuanming Ma^a^, Jianguo Feng^a^

^a^*Micro Electromechanical System Research Center of Engineering and Technology of Anhui Province, School of Microelectronics, Hefei University of Technology, Hefei, Anhui 230009, People’s Republic of China.*

*Corresponding author: *Email:* [*gbxu@hfut.edu.cn*](mailto:gbxu@hfut.edu.cn)*, Tel: +86-0551-62902263*

Fig. S1 illustrates the preparation method for PDMS/Si_3_N_4_, n-type and p-type thermoelectric (TE) materials. The corresponding materials were mixed using electric or magnetic stirring for 10 mins. Fig. S1(a) illustrates the fabrication process of the PDMS/Si₃N₄ composite, while Fig. S1(b) and (c) show the fabrication processes of the p-type and n-type thermoelectric materials, respectively.


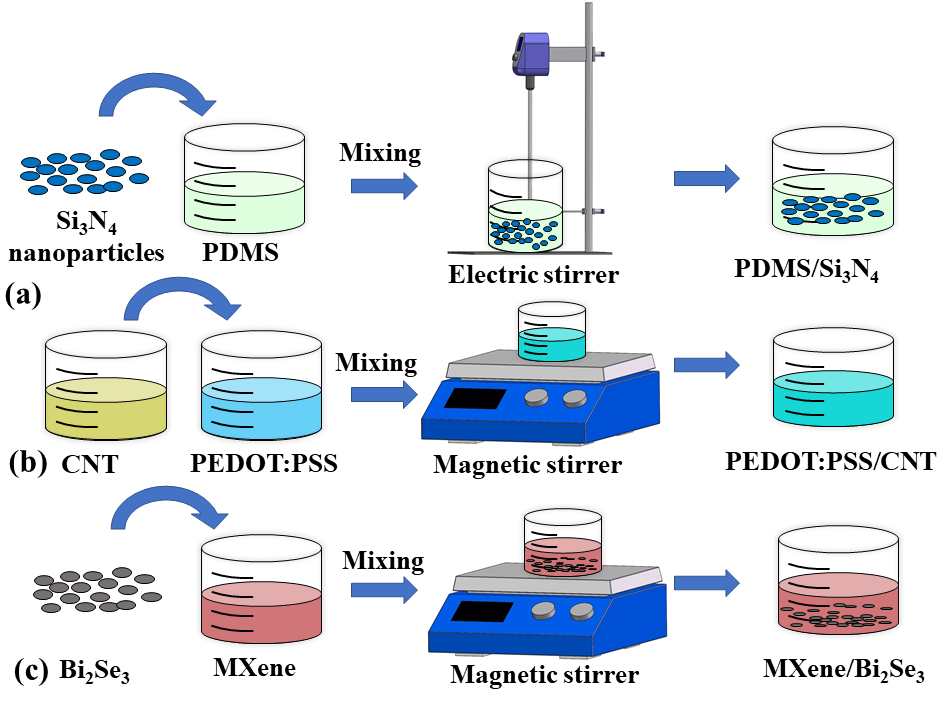


Fig. S1 Preparation of PDMS/Si_3_N_4_, n-type and p-type TE materials.(a) PDMS/Si_3_N_4_; (b) p-type; (c) n-type.

Fig. S2 shows a schematic of the water circulation cooling system, where the presence of the cavity ensures that the cooling effect is concentrated in the cold end region.


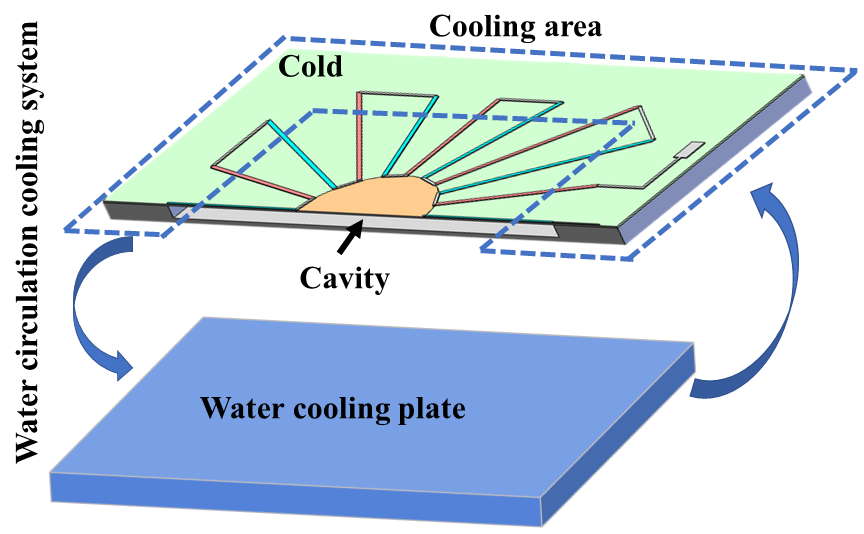


Fig. S2 Schematic diagram of the water circulation cooling system

Fig. S3 shows the performance of a single-node FTCS and an array FTCS (10 pairs) in contact mode over a temperature range of 20-100°C. Fig. S2(a) represents the single-node FTCS (the longest node in the array), with the top and bottom electrodes being MXene/Bi₂Se₃ and PEDOT:PSS/CNT, respectively. Fig. S2(b) displays the array FTCS used in this study. Fig. S2(c) shows the voltage-temperature (V-T) relationship for both sensors, measured across the 20-100°C range.


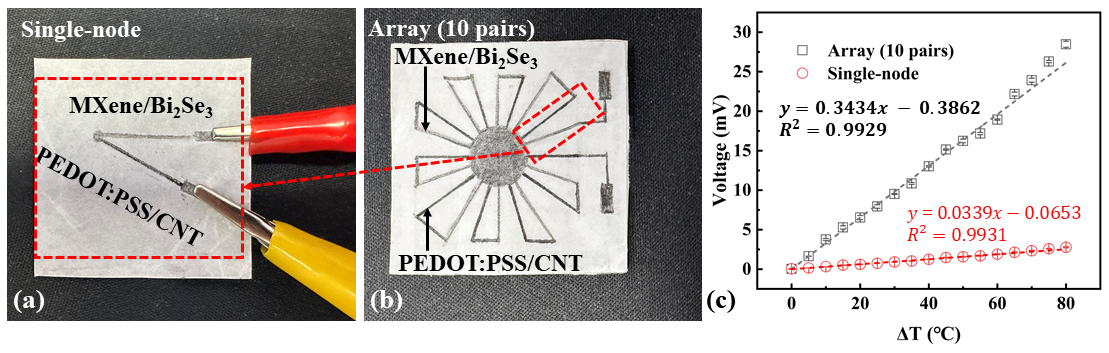


Fig. S3 The performance comparison between the single-node FTCS and the array FTCS. (a) Physical image of the single-node FTCS; (b) Physical image of the array FTCS; (c) V-T curves of both single-node and array FTCS measured in the 20-100°C range.

Fig. S4 shows the difference between a single-node and an array FTCS. Fig. S3(a) shows the single-node FTCS, while Fig. S3(b) displays the array composed of N pairs in series.


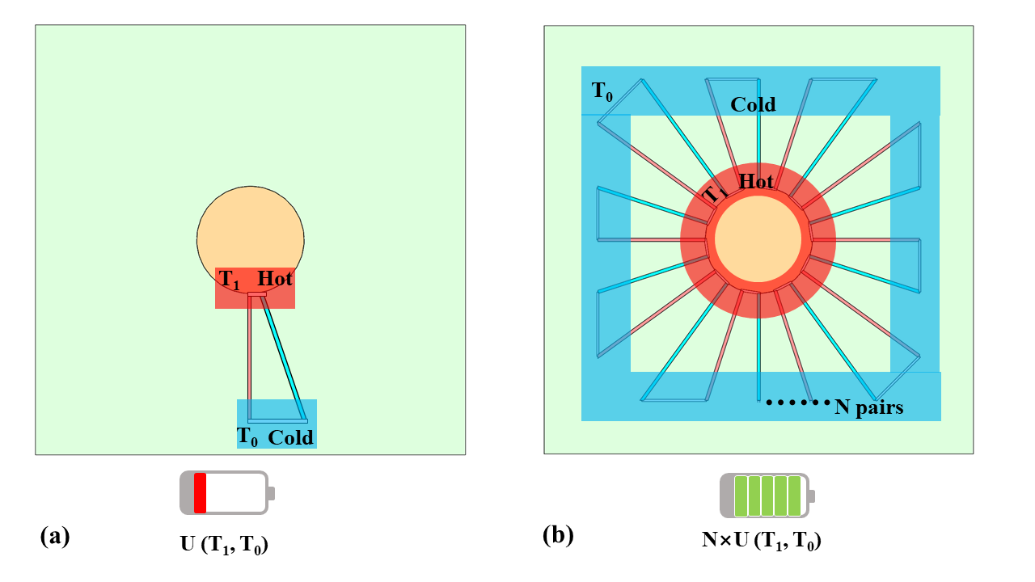


Fig. S4 Difference between a single thermocouple and an array FTCS. (a) Single-node FTCS; (b) Array FTCS composed of N pairs of thermocouples.
